# Circular RNAs promote TRPM3 expression by inhibiting hsa-miR-130a-3p in coronary artery disease patients

**DOI:** 10.18632/oncotarget.19941

**Published:** 2017-08-04

**Authors:** Ren-You Pan, Ping Liu, Hai-Tang Zhou, Wei-Xin Sun, Jun Song, Jiang Shu, Guo-Jing Cui, Zhi-Jian Yang, En-Zhi Jia

**Affiliations:** ^1^ Department of Cardiovascular Medicine, Yancheng TCM Hospital Affiliated to Nanjing University of Chinese Medicine, Yancheng 224000, Jiangsu Province, China; ^2^ Department of Cardiovascular Medicine, The First Affiliated Hospital of Nanjing Medical University, Nanjing 210029, Jiangsu Province, China

**Keywords:** microRNA, coronary heart disease, circRNA, ceRNA

## Abstract

We investigated the differential expression of circular RNAs (circRNAs) in plasma samples from three coronary artery disease (CAD) patients to identify putative therapeutic targets. We identified 24 differentially expressed circRNAs (18 up-regulated and 6 down-regulated) and 7 differentially expressed mRNAs (6 up-regulated and 1 down-regulated) in CAD patients based on competing endogenous RNA (ceRNA) microarray analysis. MiR-221(*p* = 0.001), miR-155(*p* = 0.049), and miR-130a (*p* = 0.001) were downregulated in CAD patients based on qRT-PCR analysis of another independent population of 932 study subjects (648 CAD subjects and 284 controls). We constructed a hsa-miR-130a-3p-mediated circRNA-mRNA ceRNA network using the miRanda database. This included 9 circRNAs (hsa_circ_0089378, hsa_circ_0083357, hsa_circ_0082824, hsa_circ_0068942, hsa_circ_0057576, hsa_circ_0054537, hsa_circ_0051172, hsa_circ_0032970, and hsa_circ_0006323) and 1 mRNA (transient receptor potential cation channel subfamily M member 3 [TRPM3]). We have shown that 9 circRNAs promote TRPM3 expression by inhibiting hsa-miR-130a-3p in CAD patients.

## INTRODUCTION

Cardiovascular disease (CVD) is the leading cause of human morbidity and mortality worldwide. According to World Health Organization (WHO), approximately 17.5 million people die annually from CVD [1]. This represents 31% of all global deaths. Stroke and coronary artery disease (CAD) are the major causes of CVD related deaths [1]. In China, CVD accounts for 300 deaths out of every 100,000 individuals [2], while, nearly a quarter of the Western population suffers from CAD and stroke [3–4]. Therefore, it is essential to identify risk factors associated with CVD that could be used for diagnosis and treatment at an early stage. The role of non-coding RNAs (ncRNAs) has been recognized in various human pathologies and they represent great potential as CAD biomarkers.

Human genome sequencing shows that although only 3% of the human genome codes for proteins, 80% of the human genome are transcribed [5]. The numbers of non-coding transcripts greatly exceed protein-coding mRNAs and represent species complexity [6–7]. Long noncoding RNAs (lncRNAs) are noncoding RNAs that are longer than 200 nucleotides that regulate diverse cellular functions. Recently, lncRNAs have been detected in human plasma, which can be used as disease biomarkers [8]. Aberrant levels of lncRNAs have also been reported in plasma from CAD patients [9]. Circular RNAs (circRNAs) are another class of non-coding RNAs that regulate gene expression in eukaryotes [10]. The circRNAs are competing endogenous RNAs (ceRNAs) that act as a sponge for microRNAs (miRNAs) by complementary base paring and therefore regulate gene transcription [11–12]. However, the expression and biological functions of ceRNAs in CAD are unknown. Therefore, in the present study, we generated plasma ceRNA expression profiles of three pairs of CAD and control samples to investigate their potential role as diagnostic markers in CAD.

## RESULTS

### Differentially expressed plasma circRNAs and mRNAs in CAD patients

**Figure 1:**
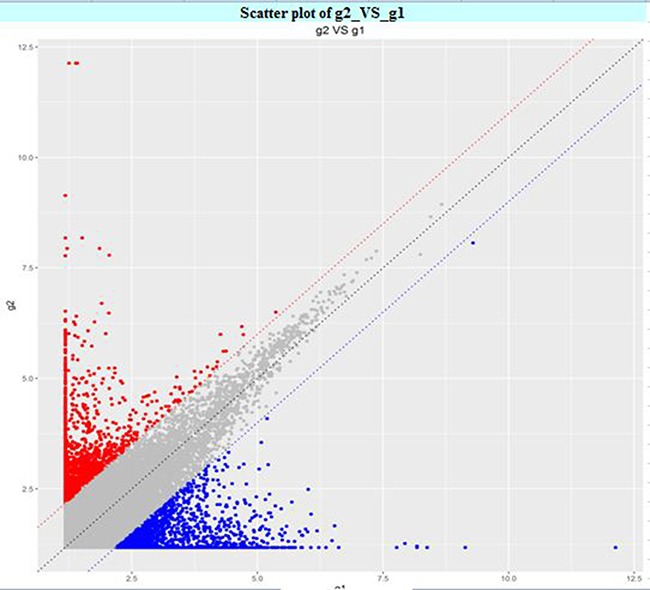
Scatter plot of differentially expressed plasma circRNAs in CAD and control subjects The X and Y axis represent average signal values (log2 scale) of plasma samples from CAD and control subjects.

**Figure 2:**
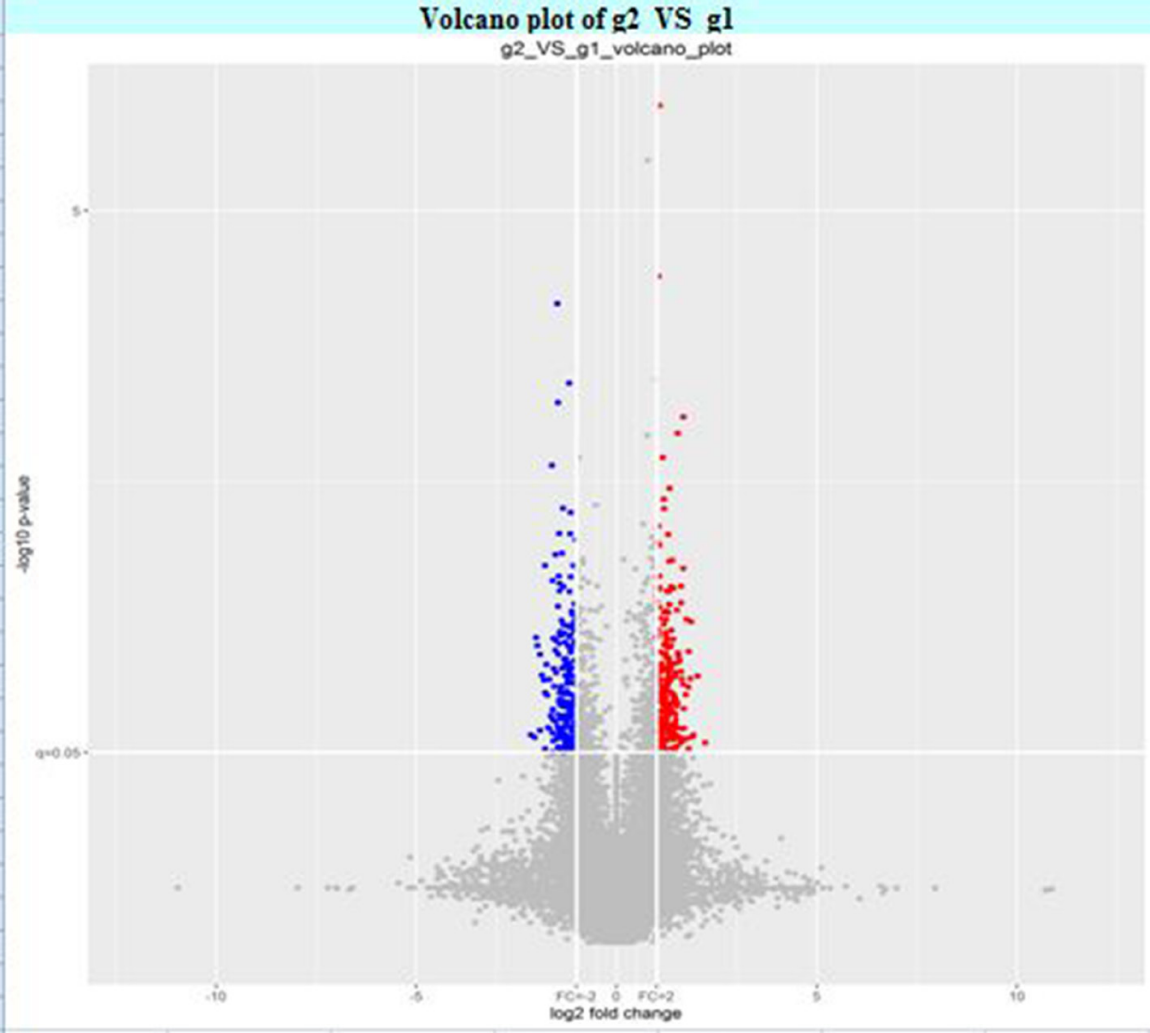
Volcano plot of the differentially expressed plasma circRNAs in CAD and control subjects

**Figure 3:**
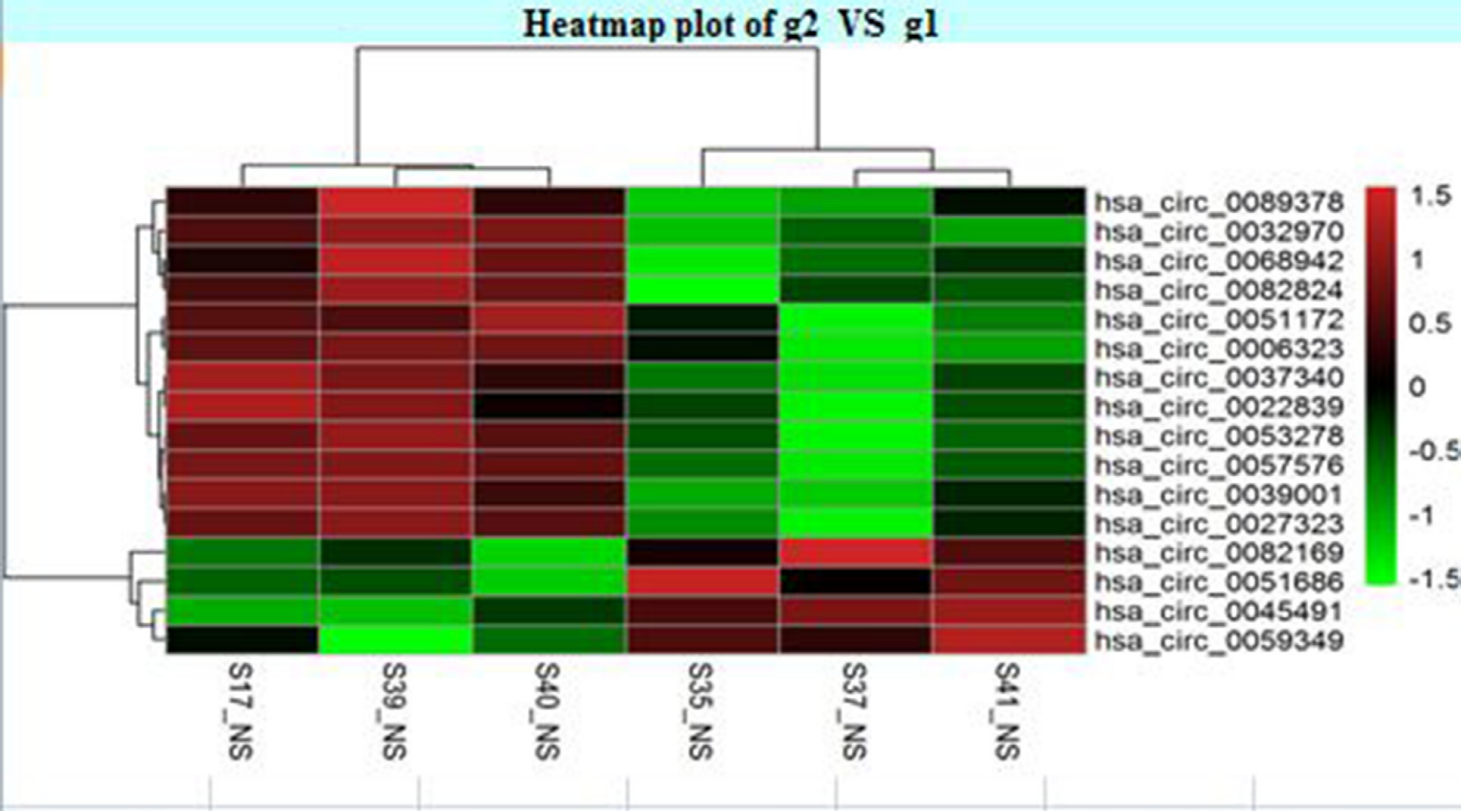
Plasma circRNA profile of CAD and control subjects Heat map shows the cirRNAs with > 1.5 fold changes. The cirRNAs are hierarchically clustered on the y-axis based on their expression. The expression index is color coded with green indicating downregulation and red indicating upregulation.

The circRNA expression profiles in human CAD and control plasma were compared by the scatter plot (Figure 1) and volcano plot filtering (Figure 2) to identify differentially expressed circRNAs. We identified 24 differentially expressed circRNAs (fold change ≥ 1.5 and *P* < 0.05) between CAD and control plasma (Table 1). Figure 3 shows the heat map of these 24 differentially expressed circRNAs. Among these, 18 circRNAs were up-regulated and 6 circRNAs were down-regulated in CAD plasma. Among the up-regulated circRNAs, 17 were exonic, and 1 intragenic. Among the down-regulated circRNAs, 6 were exonic and 1 intronic. The circRNA, hsa_circ_0051686 was both intronic and exonic. SBC Human (4*180 K) ceRNA microarray profiling showed 6 up-regulated and 1 down-regulated mRNAs (fold change ≥ 1.5 and *P* < 0.05) in CAD patients (Table 2).

**Table 1:** List of deregulated cirRNAs in 3 CAD patients

circRNAs	*p*-value	Fold change (CAD/Control)	Regulation	circRNA_ID	chromosome	gene_symbols
**hsa_circ_0082169**	0.039772045	4.122889654	down	hsa_circ_0082169	chr7	RBM28
**hsa_circ_0063721**	0.045825944	1.298228361	down	hsa_circ_0063721	chr22	KIAA0930
**hsa_circ_0033974**	0.013049935	1.958659858	up	hsa_circ_0033974	chr14	None
**hsa_circ_0026666**	0.031019957	1.79233974	up	hsa_circ_0026666	chr12	MAP3K12
**hsa_circ_0039001**	0.018213071	2.365089746	up	hsa_circ_0039001	chr16	PPP4C
**hsa_circ_0027323**	0.036944716	2.581094502	up	hsa_circ_0027323	chr12	CDK4
**hsa_circ_0083357**	0.008380919	1.920146911	up	hsa_circ_0083357	chr8	CTSB
**hsa_circ_0038998**	0.028026625	1.645027714	up	hsa_circ_0038998	chr16	PPP4C
**hsa_circ_0051686**	0.046998952	2.148530811	down	hsa_circ_0051686	chr19	MEIS3
**hsa_circ_0089378**	0.046800946	2.205308262	up	hsa_circ_0089378	chr9	VAV2
**hsa_circ_0032970**	0.001427116	2.071558488	up	hsa_circ_0032970	chr14	TC2N
**hsa_circ_0037340**	0.012987448	2.053372933	up	hsa_circ_0037340	chr16	EME2
**hsa_circ_0068942**	0.028198329	2.046034622	up	hsa_circ_0068942	chr4	ADD1
**hsa_circ_0080259**	0.018189514	1.831242935	up	hsa_circ_0080259	chr7	PSPH
**hsa_circ_0022839**	0.034785158	2.41623517	up	hsa_circ_0022839	chr11	SSSCA1
**hsa_circ_0045491**	0.008242488	4.027899063	down	hsa_circ_0045491	chr17	ARSG
**hsa_circ_0051172**	0.030127874	2.797573948	up	hsa_circ_0051172	chr19	AXL
**hsa_circ_0059349**	0.048466294	2.447186366	down	hsa_circ_0059349	chr20	PRNP
**hsa_circ_0054537**	0.014938614	1.983846543	up	hsa_circ_0054537	chr2	PSME4
**hsa_circ_0006323**	0.047365052	3.494456249	up	hsa_circ_0006323	chr1	DPYD
**hsa_circ_0082824**	0.025375501	2.440902767	up	hsa_circ_0082824	chr7	CUL1
**hsa_circ_0028926**	0.043763361	1.594160744	down	hsa_circ_0028926	chr12	ACADS
**hsa_circ_0053278**	0.021506782	2.140743547	up	hsa_circ_0053278	chr2	IFT172
**hsa_circ_0057576**	0.020268992	3.322760286	up	hsa_circ_0057576	chr2	HECW2

**Table 2:** List of deregulated mRNAs in 3 CAD patients

mRNAs	*p*-value	Fold change (CAD/Control)	Regulation	Accession	Source	chr
**LNCV6_130905_PI430048170**	0.030290601	1.886938464	up	NM_000407	RefSeq	chr22
**LNCV6_136795_PI430048170**	0.041713357	2.873491196	up	NM_078471	RefSeq	chr17
**LNCV6_104643_PI430048170**	0.005310873	2.792598516	up	NM_001007471	RefSeq	chr9
**LNCV6_141962_PI430048170**	0.043928362	1.590466991	up	NM_015136	RefSeq	chr3
**LNCV6_98175_PI430048170**	0.005752999	1.65921765	up	NM_007129	RefSeq	chr13
**LNCV6_128876_PI430048170**	0.040154272	1.743659752	up	NM_001270422	RefSeq	chr17
**LNCV6_127855_PI430048170**	0.022046703	2.447990837	down	NM_001289088	RefSeq	chr1

### Differentially expressed miRNAs in CAD

To establish a circRNA-miRNA-mRNA ceRNA network, we compared plasma miRNA expression profiles of CAD and control subjects in another independent population that was previously reported [13]. We identified 9 CAD-related miRNAs including miR-122, miR-133b, miR-214, miR-21, miR-106a, miR-130a, miR-155, miR-221, and miR-125b. Among these, miR-221 (*p* = 0.001), miR-155 (*p* = 0.049), and miR-130a (*p* = 0.001) were downregulated in CAD subjects than in non-CAD subjects (Table 3).

**Table 3:** Differentially expressed miRNAs in the 2nd set of CAD patients

Characteristics	CADs (*n* = 648)	Controls (*n* = 284)	Mann-Whitney U	*P* value
miR-125b	0.03 (0.00–0.23)	0.04 (0.00–0.27)	90214.00	0.615
miR-122	0.17 (0.00–1.82)	0.29 (0.01–1.76)	87021.50	0.182
miR-214	0.00 (0.00–0.24)	0.01 (0.00–0.26)	88581.50	0.333
miR-133b	0.15 (0.00–0.45)	0.19 (0.00–0.44)	89564.00	0.504
miR-221	0.09 (0.03–0.17)	0.12 (0.05–0.26)	79349.00	0.001
miR-21	1.95 (0.60–4.76)	1.69 (0.64–4.20)	91569.00	0.906
miR-155	151.17 (43.41–2557.31)	246.43 (65.91–2812.24)	84587.50	0.049
miR-106a	1.48 (0.74–2.98)	1.62 (0.87–3.13)	85901.50	0.106
miR-130a	2.97 (1.44–5.24)	3.48 (1.71–11.57)	79968.00	0.001

CAD, coronary artery disease. The value of each miRNAs means the relative mount calculated by 2^−Δct^ method.

### MicroRNA-mRNA interaction

The hsa-miR-221-3p, hsa-miR-155-5p, and hsa-miR-130a-3p were selected for integrated analysis of miRNA and mRNA profiling data. The miRNA target predictions were based on miRanda (release August 2010; http://www.microrna.org/). We performed integrated analysis of the inverse relations of expressed miRNAs and mRNAs in conjunction with their predicted targets and identified transient receptor potential cation channel subfamily M member 3 (TRPM3) as a target gene for hsa-miR-130a-3p.

### CircRNA-microRNA interactions

Recently, circRNAs have been identified as miRNA sponges that regulate gene expression. Therefore, we used miRanda database to investigate potential miRNAs that bind to circRNAs in CAD patients. We observed that hsa-miR-221-3p, hsa-miR-155-5p, and hsa-miR-130a-3p were bound by 10, 12, and 9 circRNAs, respectively (Table 4).

**Table 4:** MicroRNA-circRNA interactions in CAD

NO.	miRNA	miRNAup/down regulation	circRNA	circRNA up/down regulation
1	hsa-miR-221-3p	down	hsa_circ_0039001	up
2	hsa-miR-221-3p	down	hsa_circ_0083357	up
3	hsa-miR-221-3p	down	hsa_circ_0038998	up
4	hsa-miR-221-3p	down	hsa_circ_0089378	up
5	hsa-miR-221-3p	down	hsa_circ_0032970	up
6	hsa-miR-221-3p	down	hsa_circ_0068942	up
7	hsa-miR-221-3p	down	hsa_circ_0051172	up
8	hsa-miR-221-3p	down	hsa_circ_0054537	up
9	hsa-miR-221-3p	down	hsa_circ_0006323	up
10	hsa-miR-221-3p	down	hsa_circ_0082824	up
11	hsa-miR-155-5p	down	hsa_circ_0026666	up
12	hsa-miR-155-5p	down	hsa_circ_0083357	up
13	hsa-miR-155-5p	down	hsa_circ_0038998	up
14	hsa-miR-155-5p	down	hsa_circ_0089378	up
15	hsa-miR-155-5p	down	hsa_circ_0032970	up
16	hsa-miR-155-5p	down	hsa_circ_0068942	up
17	hsa-miR-155-5p	down	hsa_circ_0051172	up
18	hsa-miR-155-5p	down	hsa_circ_0054537	up
19	hsa-miR-155-5p	down	hsa_circ_0006323	up
20	hsa-miR-155-5p	down	hsa_circ_0082824	up
21	hsa-miR-155-5p	down	hsa_circ_0053278	up
22	hsa-miR-155-5p	down	hsa_circ_0057576	up
23	hsa-miR-130a-3p	down	hsa_circ_0083357	up
24	hsa-miR-130a-3p	down	hsa_circ_0089378	up
25	hsa-miR-130a-3p	down	hsa_circ_0032970	up
26	hsa-miR-130a-3p	down	hsa_circ_0068942	up
27	hsa-miR-130a-3p	down	hsa_circ_0051172	up
28	hsa-miR-130a-3p	down	hsa_circ_0054537	up
29	hsa-miR-130a-3p	down	hsa_circ_0006323	up
30	hsa-miR-130a-3p	down	hsa_circ_0082824	up
31	hsa-miR-130a-3p	down	hsa_circ_0057576	up

### Construction of ceRNA network

**Figure 4:**
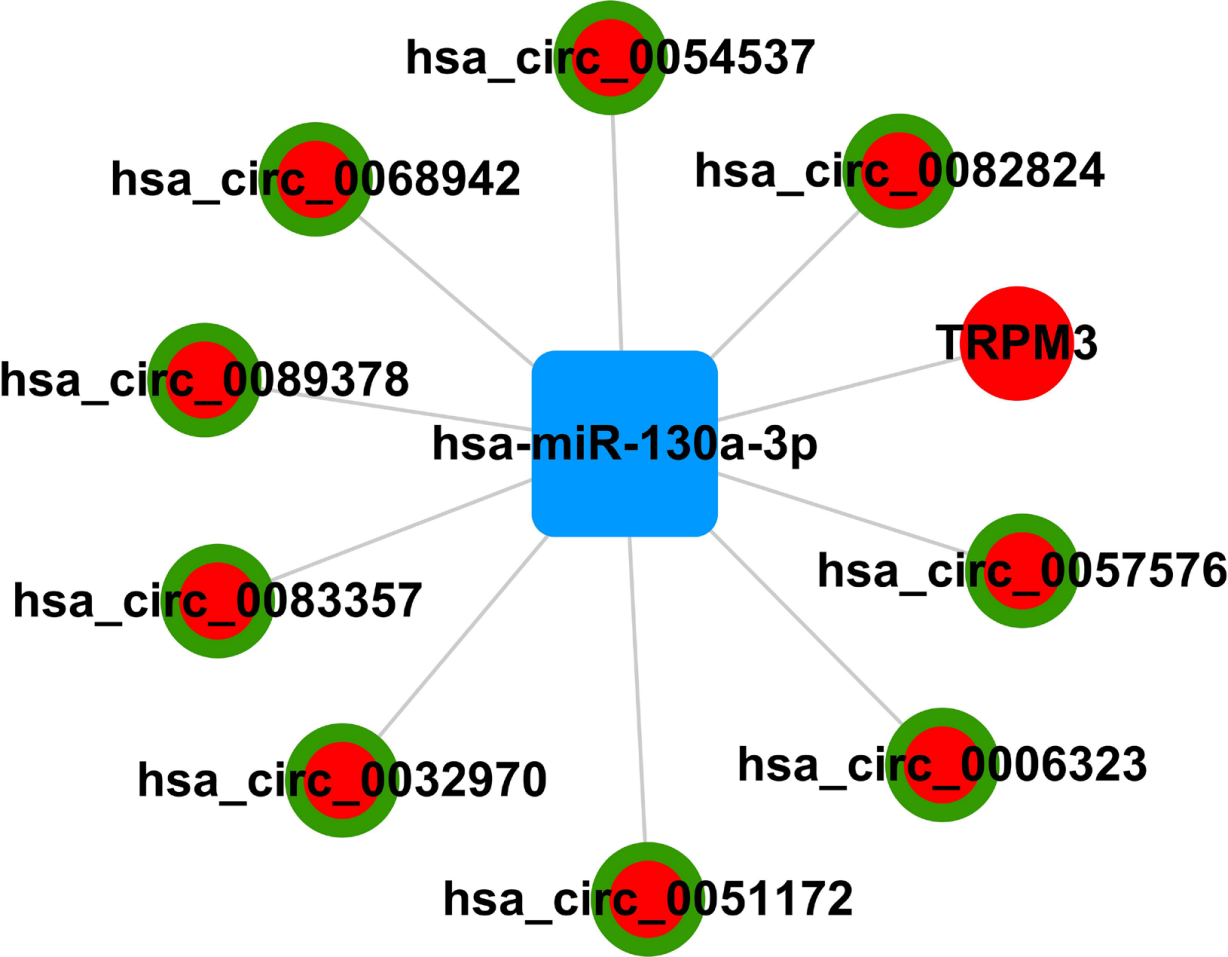
Schematic representation of hsa-miR-130a-3p-mediated circRNA-mRNA ceRNA network

Since circRNAs interact with miRNAs through miRNA response elements (MREs), we searched for putative hsa-miR-130a-3p MREs in the circRNAs using miRanda. We identified 9 circRNAs that had hsa-miR-130a-3p binding sites and negatively associated with hsa-miR-130a-3p. These included hsa_circ_0089378, hsa_circ_0083357, hsa_circ_0082824, hsa_circ_0068942, hsa_circ_0057576, hsa_circ_0054537, hsa_circ_0051172, hsa_circ_0032970, and hsa_circ_0006323. Based on these data, we constructed a hsa-miR-130a-3p-mediated circRNA-mRNA ceRNA network with 9 circRNAs and 1 mRNA (Figure 4 and Table 5).

**Table 5:** Hsa-miR-130a-3p-mediated circRNA-mRNA ceRNA network

Name	up/down regulation	Degree	ceRNA type
**hsa-miR-130a-3p**	down	10	miRNA
**TRPM3**	up	1	mRNA
**hsa_circ_0089378**	up	1	CircRNA
**hsa_circ_0083357**	up	1	CircRNA
**hsa_circ_0082824**	up	1	CircRNA
**hsa_circ_0068942**	up	1	CircRNA
**hsa_circ_0057576**	up	1	CircRNA
**hsa_circ_0054537**	up	1	CircRNA
**hsa_circ_0051172**	up	1	CircRNA
**hsa_circ_0032970**	up	1	CircRNA
**hsa_circ_0006323**	up	1	CircRNA

## DISCUSSION

The ceRNAs play a key role in post-transcriptional regulation and have been implicated in cardiovascular disease [12, 14–15]. In our previous study, we reported several miRNAs associated with CAD, but their actions were unknown [13]. In order to deciphere the ceRNA mechanisms related to CAD, we constructed a global ceRNA-miRNA-mRNA triple network using the miRanda database. We identified 9 circRNAs and 1 mRNA that formed a network with hsa-miR-130a-3p. This study sheds new insights to exploring the complex post-transcriptional regulatory networks via ceRNA interactions and identifying pathways that are altered in CAD. Subsequently, specific ceRNAs can be therapeutically used to modulate specific pathways that are involved in CAD pathology.

The miR-130 precursor is a small non-coding RNA that has been identified in mice (MI0000156, MI0000408), humans (MI0000448, MI0000748) and a range of vertebrate species (MIPF0000034). Mature miR-130 is generated by the dicer enzyme upon excision from the 3′ arm of the hairpin. MiR-130a-3p is inversely associated with coronary atherosclerosis with its down-regulation contributing to endothelial progenitor cell dysfunction in subjects suffering from coronary artery disease [16–18]. Therefore, miR-130a-3p has therapeutic potential for the prevention and treatment of CAD. Its regulation by ceRNAs as shown in this study represents one possible mode of regulation that is clinically applicable.

TRPM3 belongs to the family of transient receptor potential (TRP) channels that regulate cellular calcium homeostasis. TRPM3 mediates calcium entry potentiated by calcium store depletion. Alternatively spliced transcript variants encoding different isoforms of TRPM3 have also been identified. TRPM3 regulates proliferation and contractility of vascular smooth muscle cells in co-ordination with cholesterol and is potentially involved in therapeutic vascular modulation [19]. In the present study, we identified TRPM3 mRNA as a hsa-miR-130a-3p target and is upregulated in CAD subjects.

Circular RNAs (circRNAs) are new members of ceRNAs that are involved in regulating gene expression [20]. However, their role in CAD pathogenesis has not been reported. In the present study, we identified 24 aberrantly expressed circRNAs (18 up-regulated and 6 down-regulated) in CAD patients. These included 9 circRNAs,namely, hsa_circ_0089378, hsa_circ_0083357, hsa_circ_0082824, hsa_circ_0068942, hsa_circ_0057576, hsa_circ_0054537, hsa_circ_0051172, hsa_circ_0032970, and hsa_circ_0006323, which sponge hsa-miR-130a-3p that regulates TRPM3. This suggests that the cohort of circRNAs negatively regulate miR-130A-3p, thereby resulting in upregulation of TRPM3.

This study has several limitations that need to be addressed while interpreting our results. First, the ceRNA microarray was based on a small group of 3 CAD and 3 control subjects. Since the expression of circRNA and ceRNA can vary in individuals due to a number of factors, the circRNA and mRNA profiles were verified in another independent cohort of 932 subjects by qRT-PCR. The *in vivo* relevance of our findings requires further comprehensive investigation. Second, the mechanism of circRNA regulation of hsa-miR-130a-3p and TRPM3 is based on the bioinformatics analysis and needs to be tested in *in vitro* and *in vivo* models.

In conclusion, we identified a network of 9 circRNAs that regulate TRPM3 expression by inhibiting hsa-miR-130a-3p in CAD patients.

## MATERIALS AND METHODS

### Study subjects

In the first group, we enrolled 3 CAD and 3 control (4 males and 2 females) subjects at the Yancheng TCM Hospital Affiliated to Nanjing University of Chinese Medicine in China. The study was performed as approved by the ethics committee of the Yancheng TCM Hospital Affiliated to Nanjing University of Chinese Medicine and the First Affiliated Hospital of Nanjing Medical University. All subjects provided written informed consent. The characteristics of the study subjects are shown in Table 6.

**Table 6:** Characteristics of the CAD study population

Characteristics	CAD (*N* = 3)	Control (*N* = 3)	Total
Age (years)	64.67 ± 10.02	49.00 ± 2.65	56.83 ± 10.80
Sex (male/female)	2/1	2/1	4/2
**Physical data**			
Heart rate (bpm.)	71.33 ± 1.15	80.00 ± 20.00	75.67 ± 13.53
Height (cm)	160 ± 7	167 ± 7	164 ± 7
BMI (kg/m^2^)	26.97 ± 3.41	24.60 ± 4.18	25.55 ± 3.65
**Historical data**			
Diabetes mellitus (Y/N)	0/3	0/3	0/6
Arterial hypertension	3/0	0/3	3/3
Dyslipidaemia (Y/N)	3/0	1/2	4/2
Family history (Y/N)	0/3	0/3	0/6
**Laboratory data**			
Glucose (mM)	5.45 ± 0.28	6.48 ± 2.53	5.97 ± 1.71
TC (mM)	4.58 ± 0.39	4.10 ± 0.61	4.34 ± 0.53
TG (mM)	1.68 ± 0.51	1.77 ± 0.26	1.73 ± 0.37
HDL (mM)	1.00 ± 0.26	0.80 ± 0.44	0.90 ± 0.34
LDL (mM)	2.85 ± 0.46	2.46 ± 0.72	2.66 ± 0.58
ApoA1 (g/L)	1.12 ± 0.18	1.00 ± 0.22	1.06 ± 0.19
ApoB (g/L)	0.95 ± 0.08	0.85 ± 0.25	0.90 ± 1.18
T-Bil(μM)	24.33 ± 14.09	13.83 ± 1.85	19.08 ± 10.67
D-Bil (μM)	6.53 ± 3.70	4.20 ± 0.70	5.37 ± 2.70
Total protein (g/L)	67.23 ± 3.26	66.00 ± 5.02	66.62 ± 3.85
Albumin (g/L)	40.27 ± 1.38	40.50 ± 0.72	40.38 ± 0.99
Sodium (mM)	140.63 ± 1.72	141.30 ± 1.06	140.97 ± 1.33
Potassium (mM)	3.37 ± 0.39	3.95 ± 0.29	3.66 ± 0.44
Chloride (mM)	103.77 ± 1.40	105.03 ± 0.58	104.40 ± 1.18
Calcium (mM)	2.25 ± 0.09	2.28 ± 0.08	2.27 ± 0.07
Urea (mM)	5.87 ± 1.61	4.48 ± 0.76	5.18 ± 1.36
Uric acid (μM)	272.93 ± 56.42	385.57 ± 68.48	329.25 ± 83.40
RBC (10^12^/L)	4.58 ± 0.40	4.79 ± 0.57	4.69 ± 0.45
WBC (10^9^/L)	8.31 ± 2.75	5.95 ± 2.10	7.13 ± 2.54
PLT (10^9^/L)	135.67 ± 40.50	170.33 ± 92.09	153.00 ± 66.40
HGB (g/L)	144.33 ± 6.51	143.33 ± 18.50	143.83 ± 12.42
**Smoking status**			
Current (Y/N)	1/2	1/2	2/4
Former (Y/N)	0/3	0/3	0/6
Never (Y/N)	2/1	2/1	4/2
**Major epicardial vessel with > 50% stenosis**			
LAD (Y/N)	3/0	0/3	3/3
LCX (Y/N)	0/3	0/3	0/6
RCA (Y/N)	0/3	0/3	0/6
**Treatment**			
ACE-I (Y/N)	0/3	0/3	0/6
ARB (Y/N)	2/1	0/3	2/4
Beta-blocker (Y/N)	1/2	1/2	2/4
CCB (Y/N)	2/1	0/3	2/4
Diuretics (Y/N)	0/3	0/3	0/6
Statins (Y/N)	3/0	2/1	5/1
Anti-platelet therapy (Y/N)	3/0	3/0	6/0

Data are presented as mean + SD. n, numbers of patients.

CAD, coronary artery heart disease; BMI, body mass index (kg/m^2^); TC, total cholesterol; TG, triglyceride; HDL, high-density lipoprotein; LDL, low-density lipoprotein; ApoA1, apolipoprotein A1; ApoB, apolipoprotein B; T-Bil, total bilirubin; D-Bil, direct bilirubin; RBC, red blood cell; WBC, white blood cell; PLT, platelet; HGB, haemoglobin; LAD, left anterior descending; LCX, left circumflex; RCA, right coronary artery; ACEI, angiotensin-converting enzyme inhibitor; ARB, angiotensin receptor blocker; CCB, calcium channel blocker.

In the second group, 932 consecutive adult subjects (681 males and 251 females, 648 CAD subjects and 284 controls) aged 32–84 years that underwent coronary angiography for suspected or known coronary atherosclerosis was analyzed. They were part of a previous study on CAD. Subjects with spastic angina pectoris, infectious processes within 2 weeks, heart failure, adrenal dysfunction, and thyroid dysfunction were excluded from this study. The plasma miRNA levels were confirmed by qRT-PCR analysis. Circulating levels of miRNAs were quantified using the 2^−Δct^ method.

### Microarray analysis

Total RNA was extracted from the plasma of the subjects and purified using the mirVana^TM^ PARIS^TM^ kit (Cat#AM1556, Ambion, Austin, TX, USA) according to the manufacturer's instructions. The RNA integration number (RIN) was determined by an Agilent Bioanalyzer 2100 (Agilent technologies, Santa Clara, CA, USA). Total RNA (1μg) was amplified and labeled by Low Input Quick Amp WT Labeling Kit (Cat. # 5190-2943, Agilent technologies, Santa Clara, CA, USA) according to the manufacturer's instructions. Labeled cRNA were purified by RNeasy mini kit (Cat.# 74106, QIAGEN, GmBH, Germany).

The cDNA was labeled and hybridized to the human SBC-ceRNA (4×180k) Array, which can detect 88,371 circRNAs, and 18,853 coding transcripts (Circbase (88371), GENCODE v21 /Ensembl (18,100), LNCipedia v3.1 (40,621), Lncrnadb (28), Noncode v4 (2,608), UCSC (25,919) databases). We hybridized 1μg Cy3-labeled cRNA onto each slide using Gene Expression Hybridization Kit (Cat.# 5188-5242, Agilent technologies, Santa Clara, CA, US) in an hybridization oven (Cat.# G2545A, Agilent technologies, Santa Clara, CA, US) according to the manufacturer's instructions for 17 h. Then, slides were washed in staining dishes (Cat.# 121, Thermo Shandon, Waltham, MA, USA) with Gene Expression Wash Buffer Kit (Cat.# 5188-5327, Agilent technologies, Santa Clara, CA, USA) according to the manufacturer's instructions.

The slides were scanned by Agilent Microarray Scanner (Cat#G2565CA, Agilent technologies, Santa Clara, CA, USA) with default settings (Dye channel: Green; Scan resolution = 3 μm; PMT 100%; 20 bit). Raw data was extracted with Feature Extraction software 10.7 (Agilent technologies, Santa Clara, CA, USA) and normalized by Quantile algorithm and LIMMA packages in *R*. The microarray work was performed by Shanghai Biotechnology Cooperation, Shanghai, P.R. China.

### Construction of ceRNA network

**Figure 5:**
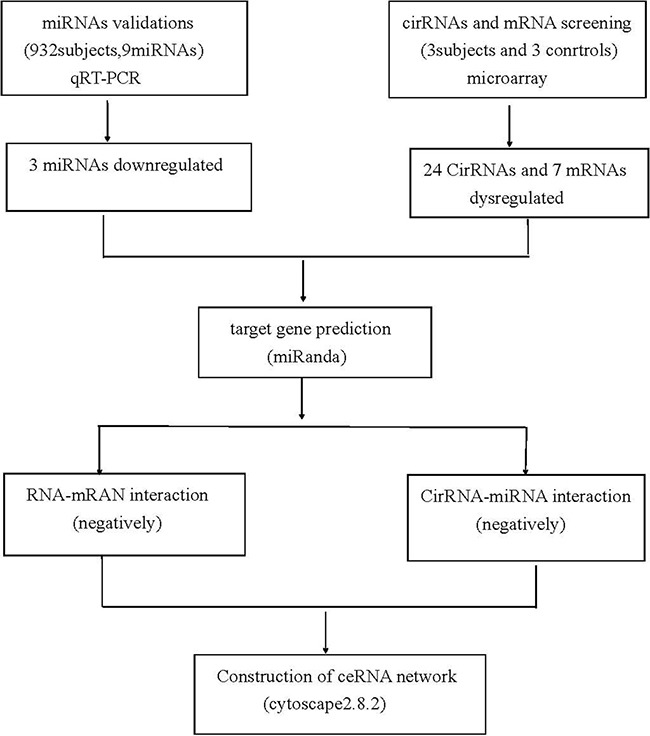
Flow chart of CAD related competing endogenous RNA network study

Figure 5 shows the methodology used to identify the ceRNA interacting genes. The miRanda method, which is based on dynamic programming (SW algorithm) and computing free energy was used to identify the target genes of miRNAs and ceRNAs [21–25]. Based on this analysis, we built a microRNA-cirRNA-mRNA interaction network. The relationship between the target and the microRNA was based on the adjacency matrix of microRNA and target A = [ai,j], where ai,j represents the weight of the relationship between the target (i) and its microRNA (j). In the microRNA-cirRNA-mRNA network, the circle represents one edge, whereas the center of the network represents a degree. The degree denotes the contribution of a microRNA to the target gene around it or the contribution of a target to the microRNAs around it. The key microRNAs and their targets in the network always had the largest degrees.
